# Data Verification and Respondent Validity for a Web-Based Sexual Health Survey: Tutorial

**DOI:** 10.2196/56788

**Published:** 2024-12-09

**Authors:** Jayelin N Parker, Theresa L Rager, Jade Burns, Okeoma Mmeje

**Affiliations:** 1 Department of Obstetrics and Gynecology, University of Michigan, 1500 E Medical Center Dr, Ann Arbor, MI, 48109, United States, 1 734-763-3429, 1 734-647-9727; 2 Department of Health Behavior and Biological Sciences, University of Michigan School of Nursing, Ann Arbor, MI, United States; 3 Department of Health Behavior and Health Education, University of Michigan School of Public Health, Ann Arbor, MI, United States

**Keywords:** sexually transmitted infections, adolescent and young adults, sexual health, recruitment, survey design, social media, data verification, web-based surveys, data integrity, social media advertisements, online advertisements, STI, STD, sexual health survey, sexually transmitted disease

## Abstract

**Background:**

As technology continues to shape the landscape of health research, the utilization of web-based surveys for collecting sexual health information among adolescents and young adults has become increasingly prevalent. However, this shift toward digital platforms brings forth a new set of challenges, particularly the infiltration of automated bots that can compromise data integrity and the reliability of survey results.

**Objective:**

We aimed to outline the data verification process used in our study design, which employed survey programming and data cleaning protocols.

**Methods:**

A 26-item survey was developed and programmed with several data integrity functions, including reCAPTCHA scores, RelevantID fraud and duplicate scores, verification of IP addresses, and honeypot questions. Participants aged 15‐24 years were recruited via social media advertisements over 7 weeks and received a US $15 incentive after survey completion. Data verification occurred through a 2-part cleaning process, which removed responses that were incomplete, flagged as spam by Qualtrics, or from duplicate IP addresses, or those that did not meet the inclusion criteria. Final comparisons of reported age with date of birth and reported state with state inclusion criteria were performed. Participants who completed the study survey were linked to a second survey to receive their incentive. Responses without first and last names and full addresses were removed, as were those with duplicate IP addresses or the exact same longitude and latitude coordinates. Finally, IP addresses used to complete both surveys were compared, and consistent responses were eligible for an incentive.

**Results:**

Over 7 weeks, online advertisements for a web-based survey reached 1.4 million social media users. Of the 20,585 survey responses received, 4589 (22.3%) were verified. Incentives were sent to 462 participants; of these, 14 responses were duplicates and 3 contained discrepancies, resulting in a final sample of 445 responses.

**Conclusions:**

Confidential web-based surveys are an appealing method for reaching populations—particularly adolescents and young adults, who may be reluctant to disclose sensitive information to family, friends, or clinical providers. Web-based surveys are a useful tool for researchers targeting hard-to-reach populations due to the difficulty in obtaining a representative sample. However, researchers face the ongoing threat of bots and fraudulent participants in a technology-driven world, necessitating the adoption of evolving bot detection software and tailored protocols for data collection in unique contexts.

## Introduction

Adolescents and young adults (AYAs) have the highest incidence of sexually transmitted infections (STIs) in the United States, in part due to their sexual behaviors, concurrent partnerships, and inconsistent condom use [1,2]. To address the burden of STIs in AYAs, their perspectives on sexual health and STIs must be appreciated. AYAs can provide insights that highlight gaps in public health knowledge and delineate youth preferences to inform health care providers and policy makers. This information will support the development of AYA-specific STI prevention resources to enhance awareness and access to testing. Understanding the factors influencing STI transmission among AYAs is critical for developing targeted interventions to curb the spread of infection and ultimately contribute to improved sexual health and well-being for this population.

Web-based surveys offer a convenient means of collecting data on STIs from AYAs, given their use of mobile technology and social media, which are suited to this tech-savvy demographic [3-6]. Through web-based surveys, individuals can confidentially share their experiences, knowledge, and behaviors related to sexual health, which is an essential consideration for AYAs who may face privacy concerns or stigma from parents and peers. Accessibility and anonymity often encourage honest and open responses, making web-based surveys a vital resource for health care professionals, researchers, and policy makers seeking to address the challenges of STIs [7].

Sexual health surveys pose recruitment challenges due to the stigma associated with sexual health behaviors [6,8]. Although participant incentives may increase survey response rates among AYAs, there is also an increased likelihood of fraudulent responses—bot, duplicate, or misrepresentative—that threaten data integrity [7,9-11]. Humans or bots may provide fraudulent responses, and the responses cannot be verified. Bots are programmed to rapidly complete surveys by posing as human respondents and threaten the integrity of web-based data collection [12-14]. Investigators must develop procedures to identify and screen out fraudulent responses as they develop their study protocols [15,16]. There is limited academic literature that describes the best practices for ensuring data verification from web-based or social media–based (eg, Facebook, Instagram) recruitment for health surveys [10,17,18]. We aim to explain the processes of ensuring data verification and a representative sample through social media recruitment for a survey-based study by sharing the steps of instrument design, programming, and data verification that resulted in our final sample.

## Methods

### Survey Design

The survey objectives were to understand AYAs’ perspectives on home-based STI testing and using virtual care for treatment (Multimedia Appendix 1). The survey was first piloted with a small group of AYAs (<20 participants) to assess for survey readability and branch logic. Respondents were then screened for eligibility using a 5-item survey. Eligible participants consented to study participation and completed both a 15-item demographic and a 26-item study survey. The surveys consisted of dichotomous, rank order, and Likert response option questions. Question domains assessed AYAs’ knowledge of STIs; privacy concerns; and preferences for testing, results, treatment communication and location, self-collection of samples, virtual care, and partner notification and referral for STI treatment.

### Survey Programming

The surveys were programmed using Qualtrics, a web-based survey tool [19]. Each survey was programmed to allow for eligibility screening and ensure data verification. Dichotomous screening questions used simple branch logic to determine eligibility. To verify age eligibility (15‐24 years), respondents were asked to enter their date of birth. Using JavaScript code, the date of birth was converted to the corresponding age based on the current date and set as embedded data (“age eligibility”). The embedded data were coded such that the age range >14 and <25 years equaled 1 and those outside this range equaled 0. Survey branching was then used to allow those with an age eligibility score of 1 to proceed with the survey. Zip codes within the 100 target counties were used to verify geographic eligibility. Eligible zip codes were compiled in a spreadsheet and verified through the Authenticate Using Contact function in Qualtrics. Embedded data (ie, county, state) were also collected for further geographic verification during data cleaning.

Several functions were incorporated to ensure data integrity against bots given that the survey was disseminated through social media. These functions, evasion techniques, and challenges are further described in Table 1. Qualtrics survey protection settings include preventing multiple submissions, reCAPTCHA scores, RelevantID fraud scores, and RelevantID duplicate scores [20-22]. IP addresses were also collected, and participants’ calculated ages based on the entered dates of birth were compared to the stated ages to prevent duplicate responses. Additional demographic questions, such as age and state of residence, were repeated throughout the survey to check for consistency. Hidden questions were implanted throughout the surveys using the JavaScript hide jQuery function in Qualtrics [10]. These honeypot questions are invisible to humans and, therefore, left blank by legitimate users. However, bots unable to read JavaScript may answer the questions, subsequently causing their survey responses to be flagged as fraudulent. Additionally, bots may fail multiple data integrity functions.

**Table 1.** Data integrity functions for web-based health surveys.^a^

Function	Source	Definition	Evasion technique	Challenges
reCAPTCHA question	Google	Provides challenges that respondents must interact with, such as selecting pictures to proceed with the survey.	Fraudulent participants can complete reCAPTCHA questions.	As bot technology grows, advanced bots may be able to select appropriate pictures to complete challenges. This was seen as bots were able to read number/letter challenges and to retype them as free-text responses to pass reCAPTCHA questions.
reCAPTCHA score	Google	Provides the probability that the respondent was a bot that was converted from a score of 0 to 1. Set as embedded data at the top of the survey flow. Scores <0.5 suggest a bot response. Scores ≥0.5 suggest a human response.	Fraudulent participants can complete reCAPTCHA questions and therefore achieve a score >0.5.	Advanced risk analysis used to determine reCAPTCHA scores relies on Google cookies. Therefore, respondents using Google products such as Chrome or Gmail are more likely to receive higher scores, suggesting a human response.
Prevention of multiple submissions	Qualtrics	Places a cookie on the respondent’s browser after they submit a response that blocks them from submitting a subsequent response when Qualtrics sees the cookie. Set as embedded data at the top of the survey flow.	Respondents can clear the browser cookies or use a different browser or device.	Participants using the same device and browser may be blocked from submitting different survey responses once the browser has a cookie placed on it.
RelevantID fraud score	RelevantID	Analyzes respondents’ browsers, operating systems, and locations to provide a fraud score of 0 to 130. Set as embedded data at the top of the survey flow. Scores ≥30 suggest a fake response.	Unable to assess, as the algorithm relies on a proprietary machine learning model.	The algorithm for the fraud score is not publicly available, and validation studies have not been completed to determine the ability to predict fraudulent responses.
RelevantID duplicate score	RelevantID	Analyzes respondents’ browsers, operating systems, and locations to provide a duplicate score from 0 to 100. Set as embedded data at the top of the survey flow. Scores ≥75 suggest a duplicate response.	Unable to assess, as the algorithm relies on a proprietary machine learning model.	Response content is not screened to determine similarity. If there is a “correct” answer to a question, and therefore expected similarity between true participants, these responses may receive a high duplicate score. Additionally, different participants using the same device may be screened out.
IP address	Qualtrics	Collects respondents’ IP addresses. Identifies duplicate IP addresses and removes them from the dataset.	Bots and fraudulent participants may use a VPN^b^ to submit multiple responses from different IP addresses.	Different respondents using the same device may be screened out.
Honeypot question	JavaScript	Hides jQuery functions that are invisible to humans. Bots may answer the hidden question as if part of the survey. Removes responses with answers to honeypot questions from the dataset.	Advanced bots may read hidden jQuery functions and therefore avoid answering questions to pose as humans.	If honeypot questions are selected to “force response,” humans will not advance to the survey.

a Various data integrity functions can be added to surveys to aid in fraudulent participant and internet bot detection. Limitations of these functions include evasion techniques used by fraudulent participants or internet bots to avoid detection, challenges in incorporating these functions into surveys, and the possibility of screening out true participants.

b VPN: virtual private network.

### Participant Recruitment

Participants in 100 US counties were recruited through Facebook and Instagram advertisements using the Meta advertising platform. These 100 counties were determined by using the Centers for Disease Control and Prevention’s 2020 STI data [23]. Five states from each US geographic region (Northeast, South, Midwest, West) with the highest chlamydia incidence in age groups 15‐19 years and 20‐24 years were identified. The five counties with the highest chlamydia incidence for both age groups were chosen within these states to promote an extensive sample recruitment. Advertisements contained the inclusion criteria and a link to the eligibility survey and were marketed with photos of demographically diverse AYAs and recruitment language such as “Researchers would like to know if individuals (15‐24 years old) would like self-administered home-based testing for sexually transmitted infections (STIs)” (Multimedia Appendix 2). Advertisement campaigns were activated for 72 hours (noon on Friday to noon on Monday) for 7 weeks (June 1, 2023, to July 24, 2023). Advertisements were targeted to all 100 selected counties for the first 3 weeks of the campaign. The geographic distribution of the responses was analyzed each week after survey completion (Tuesday through Thursday), which informed continued recruitment efforts in subsequent weeks. High response rates came from major metropolitan areas, and these were removed from the campaign in weeks 4-7 to promote recruitment from less-represented counties and aid in a more geographically diverse study sample.

### Data Cleaning

Data integrity and recruitment criteria were also verified through a 2-part cleaning process. Study-related survey responses were verified through a 6-step protocol. Study records were kept confidential, with access limited to the study team. The key linking participants’ identifications to their responses was kept in a separate file on secure software maintained by the University of Michigan, per institutional review board (IRB) protocol. First, incomplete responses were removed from the dataset. Second, responses that did not meet study inclusion criteria (eg, age, zip code, consent for screening and enrollment) were excluded. Third, responses flagged as spam by Qualtrics survey protection settings and responses that answered honeypot questions were eliminated. Fourth, duplicate IP addresses were removed. Fifth, stated and calculated ages (based on the entered dates of birth) were screened for consistency. Finally, respondents’ reported states were cross-checked against recruitment locations to verify that data met recruitment requirements.

Per IRB and the institution-based human subjects incentive program requirements, respondents were required to complete an additional survey providing their name, mailing address, zip code, and email address to receive their US $15 incentive. The embedded incentive survey link was not unique to each study participant and was only available after completing the survey. Humans were verified with a correct response to a challenge-response reCAPTCHA question and verification of the above-noted personal identifiers. These responses were linked to those provided in the demographic survey. Responses with incomplete names and mailing addresses, duplicate IP addresses, or the same longitude and latitude were excluded from the dataset. As a final verification step, IP addresses used to complete the study and incentive surveys were compared for data integrity. Consistent responses were labeled “verified” and included in the final dataset. The incentives were sent from the institution-based human subjects incentive program to the email addresses provided by the respondents. The provided zip code served as the password to access the funds.

### Ethical Considerations

This study was approved by the University of Michigan Medical School IRB (HUM00225671). Potential participants consented to be screened for eligibility prior to taking the study survey. If eligible, participants consented to enrollment in the study. Participants younger than 18 years assented to be screened for eligibility and to enroll in the study. Given the current STI rates in AYAs, it was important to include those aged 15‐17 years in the study. After completing the survey, participants were directed to the incentive survey, where their contact information was electronically obtained to disburse their US $15 incentive. Per IRB protocol, participants’ personal identifiers, including their contact information, were kept in separate, secure files on secure institutional software platforms.

## Results

**Figure 1.**
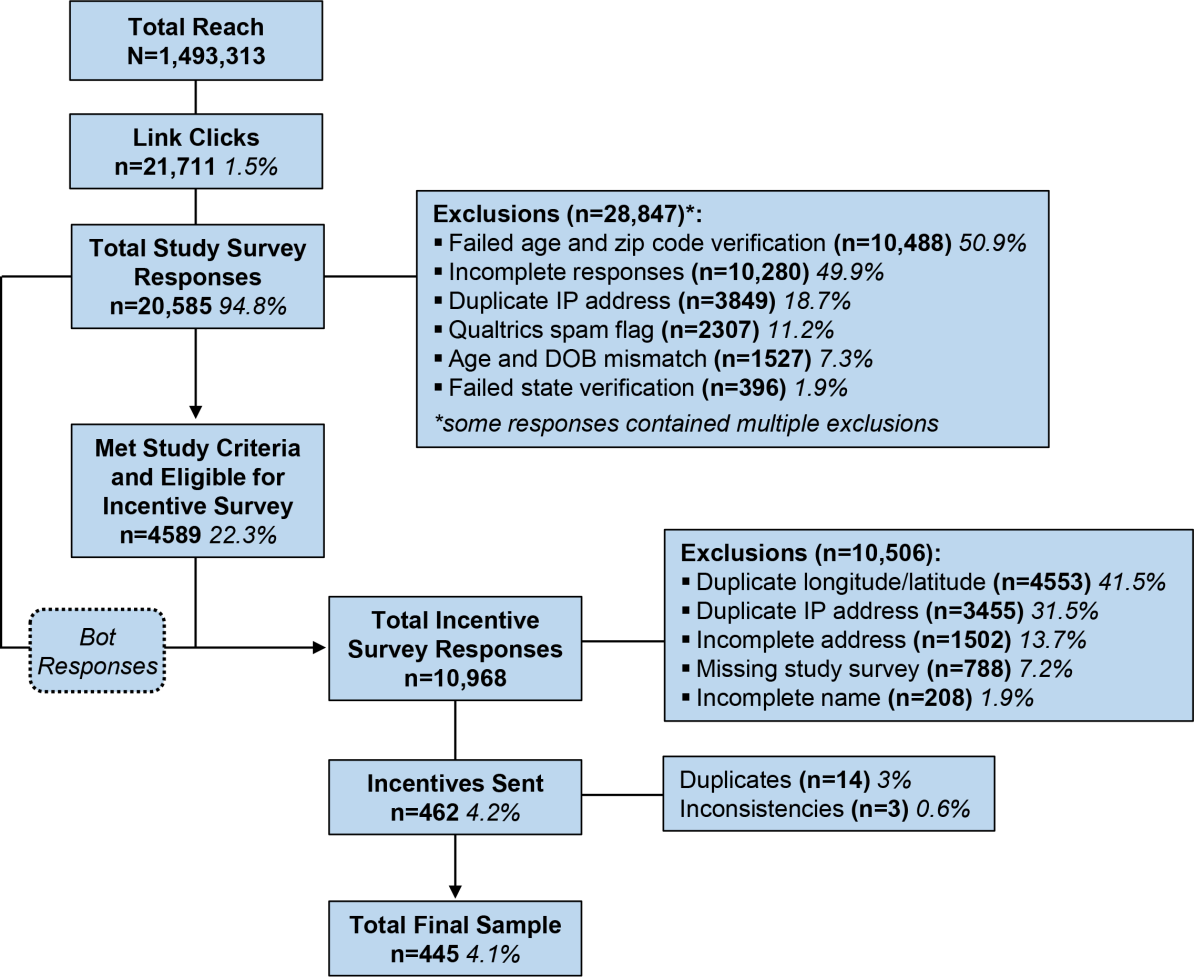
CONSORT (Consolidated Standards of Reporting Trials) diagram. DOB: date of birth.

Over 7 weeks, the social media advertisements reached 1.4 million Facebook and Instagram users in the targeted regions (Figure 1). The advertisements, which reached 1,493,313 users, received 21,711 (1.5%) total clicks, and 20,585 (94.8%) survey responses were received. Of these, 10,488 (50.9%) failed zip code and age verification, 10,280 (49.9%) were incomplete responses, 3849 (18.7%) had duplicate IP addresses, 2307 (11.2%) were flagged by Qualtrics as spam, 1507 (7.3%) failed age and date of birth verification, and 396 (1.9%) failed state verification. Of the incomplete responses, 9751 (94.9%) participants completed <25% of the survey, 197 (1.9%) completed 25%‐49%, 119 (1.2%) completed 50%‐74%, and 142 (1.4%) completed 75%‐99%. Of the 20,585 survey responses, the 4589 (22.3%) participants who met the study criteria were eligible for comparison to the incentive survey. All participants who completed the study survey were invited to complete the incentive survey, which received 10,968 responses; of these, only 462 (4.2%) were verified and sent incentives. Of those who filled out the incentive survey, 4553 (41.5%) had a duplicate longitude and latitude, 3455 (31.5%) had duplicate IP addresses, 1502 (13.7%) had incomplete addresses, 788 (7.2%) did not complete the study survey, and 208 (1.9%) had incomplete names; none of these respondents were sent incentives. Of the 462 incentives sent, 14 (3%) responses were duplicates, and 3 (0.6%) contained discrepancies, resulting in a final sample of 445 (4.1%) out of the 10,968 incentive survey responses. Our final sample included 109 (24.5%) participants from the West, 113 (25.4%) from the South, 125 (28.1%) from the Midwest, and 98 (22%) from the Northeast, demonstrating an equal distribution across the 4 regions. Most identified as White (n=291, 65.4%), non-Hispanic (n=371, 83.4%), cisgender female (n=201, 45.2%) or cisgender male (n=175, 39.3%), heterosexual or straight (n=269, 60.4%), and ages 20‐24 years (n=295, 66.3%). The total social media advertisement cost was US $2168—approximately US $0.10 per click and US $4.87 per complete response. The total cost of incentives and advertisements was US $9098.

## Discussion

### Principal Results

Despite numerous fraudulent responses, this web-based survey was a productive tool for recruiting AYAs. It was paramount to develop a robust data integrity methodology before recruitment. Although the surveys incorporated several data integrity functions, bots were able to circumvent many features, including honeypot questions and RelevantID and reCAPTCHA scores [15]. Therefore, it is important to have multiple data integrity strategies in place to identify fraudulent responses that may be detected with various verification options.

Due to the high volume of responses obtained in focused advertising over 72 hours, it was necessary to pause advertisements to review and verify responses, distribute incentives, and adjust our recruitment strategy. Studies intended to collect large survey responses may require several study staff to process responses promptly. Additionally, the continuous adaptation of our recruitment strategy was critical to obtaining representative responses from each of the 100 predetermined counties via a web-based survey. A high number of responses were fraudulent—particularly those from densely populated cities (eg, New York City, Los Angeles, Atlanta, Chicago). It is possible that Facebook and Instagram’s like, comment, and share features exaggerated recruitment from these cities, leading to a higher volume of fraudulent responses. Monitoring our sample’s demographics and recruitment progress allowed us to adapt our targeted advertisements to garner responses from as many of the 100 counties included in the study and reduce the number of responses from saturated regions. Similar to prior authors’ findings [7,9-12,15,17,18,20,24,25], our work revealed that bots are evolving and increasing the risk of fraudulent data in web-based research. As technology continues to advance, bots and artificial intelligence pose a serious risk to data integrity, and data integrity functions, such as reCAPTCHAs, may not be adequate to prevent bot responses in the future [26].

### Limitations

Limitations in our data collection process were uncovered during the data cleaning process. We noted that the same participant could submit duplicate responses using a virtual private network (VPN) with different IP addresses. VPN users can be detected by using VPN detection software or an IP database to compare IP addresses to known affiliated VPNs; however, we were only made aware of cost-effective versions of these programs after analysis. Additionally, duplicate IP addresses were not detected when responses were submitted using the same IP address in subsequent weeks, as data screening and incentive distribution occurred weekly during recruitment without comparisons across all weeks. Future studies should continuously compare all IP addresses, even if responses are verified in batches. Fraudulent response detection proved to be challenging. If different participants had submitted the survey on the same device (ie, used the same IP address), they would have been screened out of the sample. This ensured that the same participant was not submitting multiple times under different aliases; however, this method excluded participants who may have legitimately had the same IP address. Similarly, this method excluded potential legitimate participants with the same longitude and latitude. Additionally, we did not include inverse questions to further verify responses, as it was important to keep the survey short to prevent respondent fatigue; however, web-based survey data can suffer from inconsistent and invalid responses [7,27,28]. Participants were required to corroborate their age, date of birth, and zip code with the respective county to participate in the study. However, zip codes and county lines do not always neatly share boundaries, limiting our study. Incomplete responses were excluded from the study, which may have introduced bias into the results. However, of the incomplete responses, nearly half of the participants completed <2% of the survey, suggesting these respondents failed eligibility screening. As only 19 (0.2%) of the 10,280 incomplete responses were from participants who completed 98% of the survey, very few respondents failed to press save and submit, causing exclusion from analysis. Our final sample only included participants whose responses and personal identifiers were corroborated by both the study and the incentive surveys to ensure data integrity and participant validity. However, listwise deletion may have resulted in biased results [29]. The responses of those who exited the incentive survey before completion were not included in the final analysis because their responses and identities could not be verified, potentially eliminating responses from participants who did not want to participate in the incentive survey. Due to the rigor and time intensity of the data cleaning process, future researchers should consider study team capacity and refrain from advertising the study incentive when designing surveys that are subject to fraudulent responses.

### Conclusions

Confidential web-based surveys are appealing for assessing populations that are historically hard to reach—specifically AYAs, who are not comfortable disclosing their sexual behaviors and sexual and gender identities to family, friends, or clinical providers [10,30]. Web-based surveys may garner fraudulent responses from both bots and humans; nevertheless, they are invaluable to researchers. Investigators must be prepared to receive and address fraudulent responses in a tech-forward world and understand that there are tools available for detecting such responses. Investigators must identify and develop the best bot and other fraudulent response detection protocols for online data collection. As new detection software becomes available and new bots are programmed, it is critical to employ new technologies to support data integrity.

## Supplementary material

10.2196/56788 Multimedia Appendix 1 Survey questions.

10.2196/56788 Multimedia Appendix 2 Social media kit.

## Abbreviations

AYA: adolescent and young adult
IRB: institutional review board
STI: sexually transmitted infection
VPN: virtual private network
