# Supplementary material for: Data Verification and Respondent Validity for a Web-Based Sexual Health Survey: Tutorial
Source: JMIR Form Res. 2024 Dec 9;8:e56788. doi: 10.2196/56788 (PMC11648336; doi:10.2196/56788)
Supplement: Multimedia Appendix 1 [file formative-v8-e56788-s001.docx]

SoMe Survey

Screening Questionnaire

1. Are you comfortable with reading and completing study questionnaires in English text?
2. Yes
3. No
4. Have you been sexually active (i.e., vaginal and/or anal sex - penile-vaginal or penile-anal intercourse) in the last 60 (sixty) days?
5. Yes
6. No
7. Do you have regular access to social media (i.e., Facebook®, Instagram®, Twitter®)?
8. Yes
9. No
10. What social media apps do you use?
11. What is your date of birth? (MM/DD/YYYY)

Study Questionnaire

1. Have you ever been tested for a sexually transmitted infection (STI) before?
2. Yes
3. No
4. Unsure
5. Are gonorrhea and chlamydia STIs?
6. When do you think someone should get tested for an STI? Check all that apply.
7. Only if they have symptoms like abnormal discharge, pain, burning when peeing
8. After a new sexual partner
9. Every year if they are sexually active
10. If you wanted to get tested for a STI (eg, chlamydia, gonorrhea, trichomonas), how likely are you to test yourself at home with a vaginal swab or urine collection and mail it to a lab for a result?
11. Extremely unlikely
12. Somewhat unlikely
13. Neither likely nor unlikely
14. Somewhat likely
15. Extremely likely
16. If it cost the same, which would you prefer for convenient STI testing?
17. Virtual care (i.e., video or phone visit) with home-based STI testing
18. In-person STI testing at a clinic, school, doctor’s office, or pharmacy
19. If you wanted to use a home-based STI test kit, how would you like to receive it?
     Drag the responses to rank them from 1-4 with 1 (one) being most favored and 4 (four) least favored.
20. Pick up from school clinic
21. Pick up from your doctor's office
22. Pick up from local public health department
23. Mailed to your home

|  |
| --- |

1. Do you know how to order an STI test kit online?
2. Yes
3. No
4. Have you ever ordered an STI test kit from a website?
5. Yes
6. No
7. How comfortable would you be receiving a package to your home for STI testing?
8. Extremely uncomfortable
9. Somewhat uncomfortable
10. Neither comfortable nor uncomfortable
11. Somewhat comfortable
12. Extremely comfortable
13. Do you think you can have a home-based STI test kit delivered to your home without anyone else in your home seeing it?
14. Yes
15. No
16. How would you feel if someone in your home saw that you received a home-based STI test kit?
17. Extremely uncomfortable
18. Somewhat uncomfortable
19. Neither comfortable nor uncomfortable
20. Somewhat comfortable
21. Extremely comfortable
22. How comfortable are you with collecting a vaginal swab (females) or urine (males) for a home-based STI test?
23. Extremely uncomfortable
24. Somewhat uncomfortable
25. Neither comfortable nor uncomfortable
26. Somewhat comfortable
27. Extremely comfortable
28. How would you like to receive instructions on how to collect the vaginal or urine sample?
     Drag the responses to rank them from 1-4 with 1 (one) being most favored and 4 (four) least favored.
29. Written
30. Online video
31. A telephone call to speak with a person
32. A telephone number to text with a person
33. If you collected a vaginal swab or urine sample and sent it for processing, how confident would you be in the accuracy of the result?
34. Extremely unconfident
35. Somewhat unconfident
36. Neither confident nor unconfident
37. Somewhat confident
38. Extremely confident
39. If you were to complete a home-based STI test, would you return your sample to the post office or mailbox in a pre-paid return envelope?
40. Yes
41. No
42. How would you like to receive test results?
     Drag the responses to rank them from 1-5 with 1 (one) being most favored and 5 (five) least favored.
43. Text
44. Phone call
45. Logging into a website
46. Secure email
47. Letter to your home
48. If you had an STI, do you have a healthcare provider you can go to for treatment of the infection?
49. Yes
50. No
51. Do you ever talk to your friends about getting tested for an STI?
52. Yes
53. No

For the following questions a current sexual partner is anyone you have had penile-vaginal or penile-anal intercourse within the last 60 days.

1. If you were diagnosed with an STI (i.e., chlamydia, gonorrhea, trichomonas) would you tell your **current sexual partners** about virtual care (i.e., video or phone visit) with home-based STI testing and treatment?
2. Yes
3. No
4. If you were diagnosed with an STI (i.e., chlamydia, gonorrhea, trichomonas) would you tell your **current sexual partners** about in-person STI testing and treatment at a clinic, school, doctor's office, or pharmacy?
5. Yes
6. No
7. If you have more than one sex partner, would you tell **all** of them about virtual care (i.e., video or phone visit) with home-based STI testing?
8. Yes
9. No
10. If you have more than one sex partner, would you tell **all** of them about in-person STI testing and treatment at a clinic, school, doctor’s office, or pharmacy?
11. Yes
12. No
13. How comfortable are you with the idea of virtual care for STI testing?
14. Extremely uncomfortable
15. Somewhat uncomfortable
16. Neither comfortable nor uncomfortable
17. Somewhat comfortable
18. Extremely comfortable
19. Have you ever met with a doctor or health care provider virtually, that is, over the internet using a laptop, smart phone, or computer?
20. Yes
21. No
22. Do you have internet access that would allow you to meet with a doctor for virtual care with internet access on a mobile device (i.e., smartphone or computer)?
23. Yes
24. No
25. Do you have any privacy concerns related to accessing and using the internet in your home?
26. Yes
27. No

Demographics Questionnaire

1. Do you consider yourself Hispanic or Latino?
2. Yes
3. No
4. Decline to answer
5. What best describes your Hispanic or Latino ancestry? Select all that apply.
6. Cuban
7. Dominican
8. Mexican
9. Puerto Rican
10. Other
11. Decline to answer
12. What is your race? Select all that apply.
13. White
14. Black or African American
15. American Indian / Alaskan Native
16. Middle Eastern / Northern African (MENA)
17. Asian
18. Pacific Islander / Native Hawaiian
19. Other. Please specify:
20. Decline to answer
21. What is your age (in years)?
22. What state do you currently live in?
23. What is the highest level of school you completed?
24. None, no formal schooling
25. 6th - 8th grade
26. 9th - 11th grade
27. High school diploma
28. High school certificate of completion (no diploma)
29. GED
30. Some college, technical school, or vocational school
31. Technical or vocational school graduate
32. Two-year college graduate
33. Four-year college graduate
34. Some graduate school
35. Master's degree or above
36. Decline to answer
37. Are you currently employed?
38. Yes
39. No
40. Decline to answer
41. Are you employed full-time or part-time?
    Part-time means that you work less than 35 hours per week during most weeks.
42. Full-time
43. Part-time
44. Decline to answer
45. In the past 30 days, how much money have you received?
46. $0-249
47. $250-499
48. $500-749
49. $750-999
50. $1,000-1,999
51. $2,000-3,999
52. $4,000 or above
53. Unsure
54. Decline to answer
55. What is your current gender identity? Select all that apply.
    1. Cis woman
    2. Cis man
    3. Trans woman
    4. Trans man
    5. Genderqueer or gender nonconforming
    6. Non-binary
    7. Pan-gender
    8. Two Spirit
    9. Other. Please specify:
    10. Decline to answer
56. What sex were you assigned at birth?
57. Female
58. Male
59. Decline to answer
60. How would you describe your current relationship status?
61. I am single, but having sex with other people
62. I am single, and not having sex with other people
63. I am casually dating
64. I have a boyfriend or girlfriend
65. I have a partner or lover
66. Although we lack a legal commitment, I have a partner and we have had a commitment ceremony
67. I am in a civil union or domestic partnership
68. I am legally married
69. Other. Please specify:
70. How many sexual partners have you had in the past 60 days?
    1. 0
    2. 1
    3. 2
    4. 3
    5. 4
    6. 5
    7. >6
71. How long have you and your primary sexual partner been together?
    1. Less than 30 days
    2. 1-3 months
    3. 4-6 months
    4. 7 months to 1 year
    5. 1-3 years
    6. 3 years or more
    7. Decline to answer
72. In the last 5 years, who did you have sex with?
    By sex, we mean any activity you personally consider as sexual activity.
    1. Cisgender Women
    2. Cisgender Men
    3. Transgender Women / Male-to-Female (MTF)
    4. Transgender Men / Female-to-Male (FTM)
    5. I have not had sex with anyone in the last 5 years.
73. Which of the following best describes your current sexual orientation?
    1. Straight / heterosexual
    2. Asexual
    3. Aromantic
    4. Lesbian
    5. Gay
    6. Bisexual
    7. Queer
    8. Same-gender loving
    9. Other. Please specify:
    10. Decline to answer
74. Are you attracted to men, women, or both?
75. Please indicate how sexually attracted you are to the following genders.

(Not at all, Not very, Somewhat, Very, Unsure)

1. Cisgender Women
2. Cisgender Men
3. Transgender Women / Male-to-Female (MTF)
4. Transgender Men / Female-to-Male (FTM)
5. Females at birth, Genderqueer
6. Males at birth, Genderqueer

SoMe Incentive Survey

Please complete the following to receive your Visa gift card.

1. Full name (first last)
2. Please enter a valid address. Your address will not be used to mail any items, and your zip code will be required to access your gift card. Please note that entering an incorrect address may delay your ability to receive your study incentive of $15.
3. Please provide your contact email address. Your email address will be used to share your $15 gift card incentive.
4. Confirm your email address by entering it again.
