# Supplementary material for: Data Verification and Respondent Validity for a Web-Based Sexual Health Survey: Tutorial
Source: JMIR Form Res. 2024 Dec 9;8:e56788. doi: 10.2196/56788 (PMC11648336; doi:10.2196/56788)

Mmeje STI Study Social Media Advertisements
April 10, 2023

SOCIAL MEDIA AD CONTENT: To be used on Facebook and Instagram, sponsored by the main Michigan Medicine platforms. All ads will direct users to the study specific website: (insert URL here)

Potential Text:

- STI Research
- Interested in STI testing?
- We want to hear from you.
- Please take our survey!
- Take our survey!
- Researchers would like to know if individuals (15 - 24 years old) would like self-administered home-based testing for sexually transmitted infections (STIs).
- Researchers would like to know if youth (15 - 17 years old) would like self-administered home-based testing for sexually transmitted infections (STIs). No parental consent required!
- Researchers would like to know if individuals, aged 18 - 24, would like self-administered home-based testing for sexually transmitted infections (STI).
- We want to know if individuals, age 15-24, would like self-administered, home-based testing for sexually transmitted infections (STIs).
- The purpose of this survey is to understand whether home-based STI testing is an acceptable option for youth (age 15-24) seeking STI testing and treatment.
- This project aims to understand whether home-based STI testing is an acceptable option for youth seeking STI testing and treatment.
- National Perspective of Youth: Home-Based STI Testing
- National Perspective of Youth: Home-Based STI Testing is a national survey asking about 15-24 year olds perceptions of STI testing!
- By taking this paid 15-minute survey, you may help others who struggle with accessing STI testing and treatment in the future.
- U-M researchers are seeking youth, aged 15-24, to participate in a virtual survey to understand whether home-based STI testing is an acceptable option for those seeking STI testing and treatment.
- We are seeking youth, aged 15-24 to participate in a 15-minute online survey about understanding the attitudes and barriers toward home-based STI screening and treatment.
- Help us understand the attitudes and barriers toward home-based STI screening and treatment for youth (ages 15-24) by participating in this paid 15-minute survey.
- Take this paid 15-minute survey to help researchers understand the barriers and attitudes toward home-based STI testing for youth (ages 15-24).
- Earn a $15 gift card for participating!
- Tell us your thoughts by participating in our survey!
- We want to know what youth (ages 15-24) think about home-based STI testing in this 15-minute survey.
- In this paid 15-minute survey, U-M researchers hope to learn more about the attitudes and barriers toward home-based STI testing for youth, ages 15-24.
- The results of this online survey will help researchers understand if home-based STI testing and virtual care can increase future access to sexual and reproductive healthcare services.

Mmeje STI Study Social Media Advertisements
April 10, 2023

- Participating in this 15-minute survey can help understand whether home-based STI testing is acceptable to youth (age 15-24) seeking STI testing and treatment.
- Virtual Research Opportunity!
- Youth/Young adults: Tell us your thoughts!
- Home-based STI Testing Survey
- Online Survey: We want to hear from you
- No parental consent required!

Potential Images:

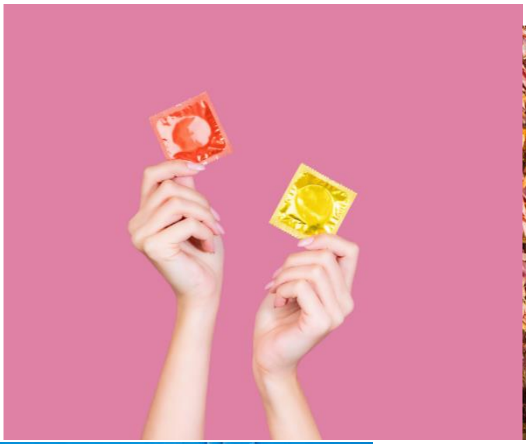
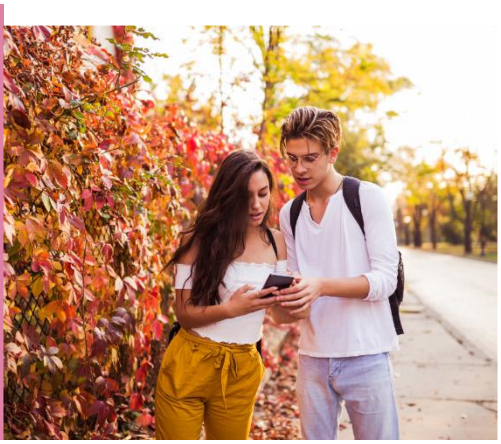
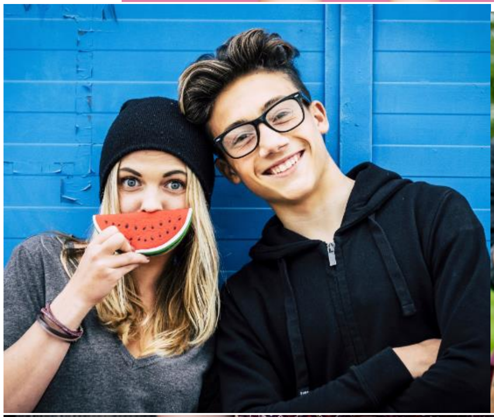
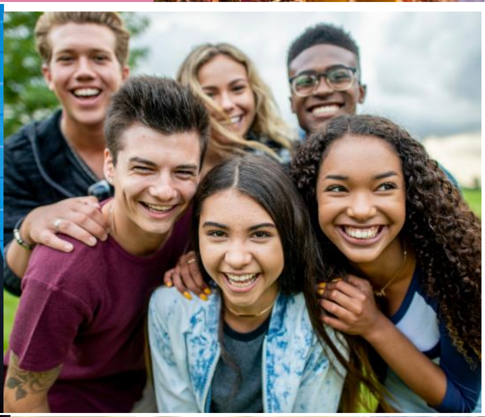
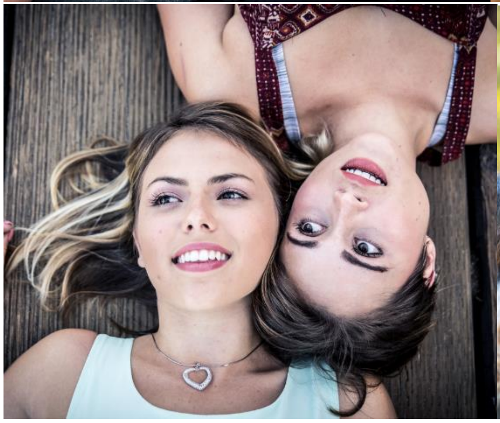
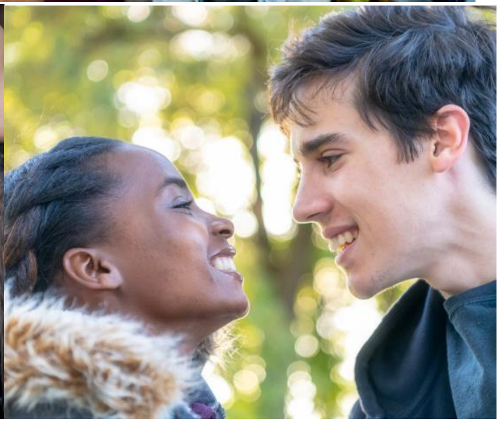

Mmeje STI Study Social Media Advertisements
April 10, 2023

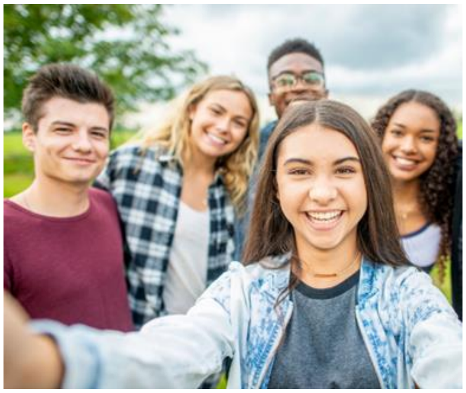

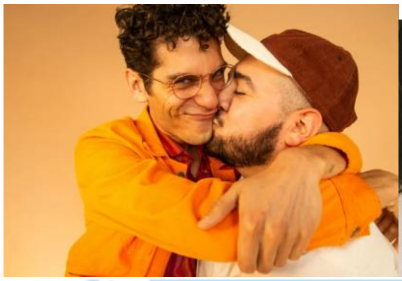

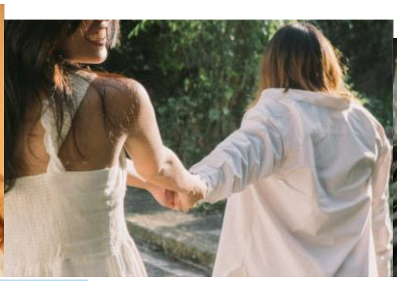

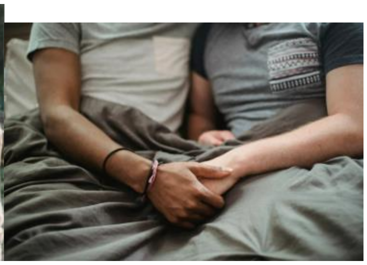

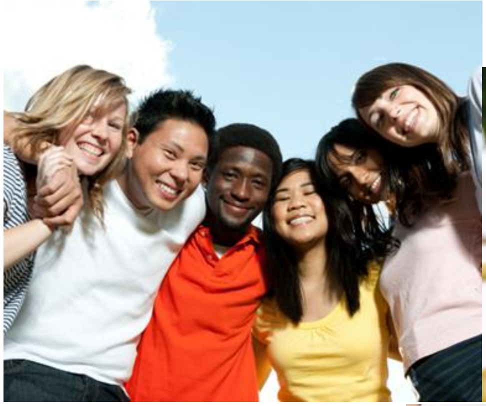

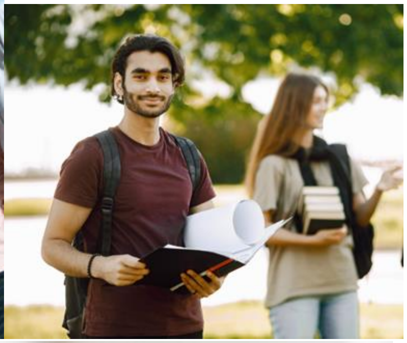

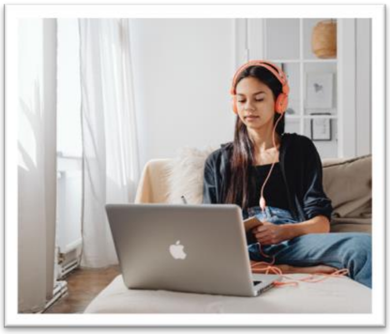

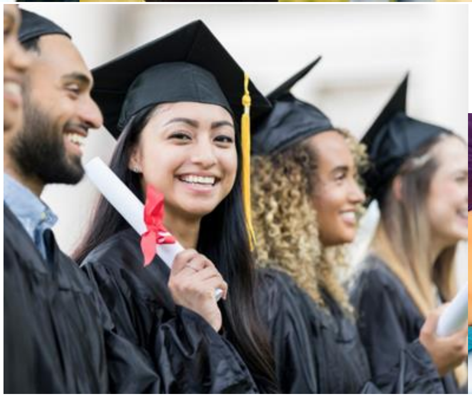

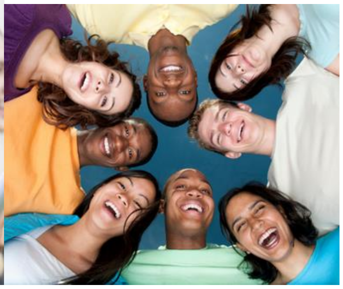

Mmeje STI Study Social Media Advertisements
April 10, 2023

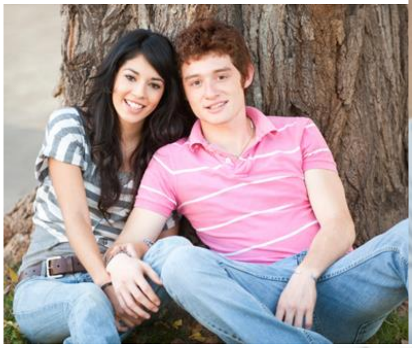
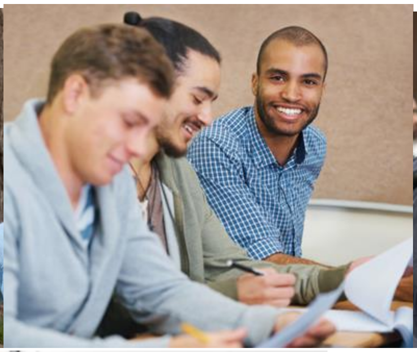
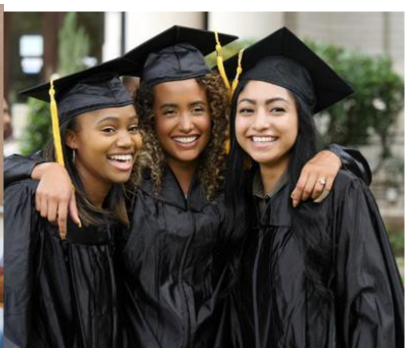

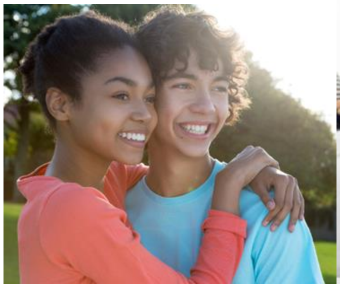
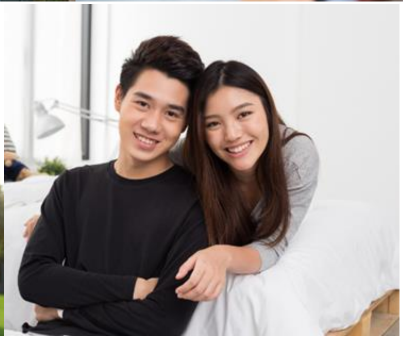

Example Ad:

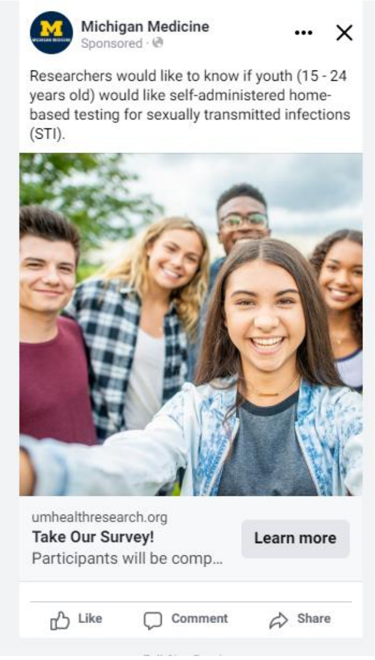

Supplement: Multimedia Appendix 2 [file formative-v8-e56788-s002.pdf]
